# A Context-Aware S-Health Service System for Drivers

**DOI:** 10.3390/s17030609

**Published:** 2017-03-17

**Authors:** Jingkun Chang, Wenbin Yao, Xiaoyong Li

**Affiliations:** 1 Beijing Key Laboratory of Intelligent Telecommunications Software and Multimedia, Beijing University of Posts and Telecommunications, Beijing 100876, China; nevious@163.com; 2 The Key Laboratory of Trustworthy Distributed Computing and Service, Ministry of Education, Beijing University of Posts and Telecommunications, Beijing 100876, China; lxyxjtu@163.com

**Keywords:** driver health, context awareness, smart health, semantic sensor network (SSN), situation assessment, customized business services

## Abstract

As a stressful and sensitive task, driving can be disturbed by various factors from the health condition of the driver to the environmental variables of the vehicle. Continuous monitoring of driving hazards and providing the most appropriate business services to meet actual needs can guarantee safe driving and make great use of the existing information resources and business services. However, there is no in-depth research on the perception of a driver’s health status or the provision of customized business services in case of various hazardous situations. In order to constantly monitor the health status of the drivers and react to abnormal situations, this paper proposes a context-aware service system providing a configurable architecture for the design and implementation of the smart health service system for safe driving, which can perceive a driver’s health status and provide helpful services to the driver. With the context-aware technology to construct a smart health services system for safe driving, this is the first time that such a service system has been implemented in practice. Additionally, an assessment model is proposed to mitigate the impact of the acceptable abnormal status and, thus, reduce the unnecessary invocation of the services. With regard to different assessed situations, the business services can be invoked for the driver to adapt to hazardous situations according to the services configuration model, which can take full advantage of the existing information resources and business services. The evaluation results indicate that the alteration of the observed status in a valid time range *T* can be tolerated and the frequency of the service invocation can be reduced.

## 1. Introduction

Safe driving is the most important topic that disturbs professional or elderly drivers in everyday life. These drivers usually suffer from various chronic diseases (e.g., hypertension, diabetes and cardiovascular disease) and accompanying symptoms which can lead to a high risk of sudden illness (e.g., heart attack, hypertensive crisis) [1,2]. Without awareness of health or environmental deterioration while driving, drivers may need a longer reaction time to handle emergencies and become easily involved in traffic accidents [3], thus posing a danger to other drivers, passengers, pedestrians and property. Accordingly, it is highly desirable for the monitoring of the drivers’ health conditions to estimate their current status and serve them with appropriate service to ensure safety and thus prevent the possibility of an accident.

Emerging sensing technologies enhance healthcare with pervasive and nonintrusive health data collection, which has been considered as a cornerstone technology for abnormality detection, intervention, and effective management [4,5]. Taking advantage of sensing, mobile computing, and communication technologies, M-health has been proven to be an effective method for improving health and health services in the form of medical recommendations, tele-monitoring, and mobile health assistance [6,7,8,9]. As a real-time, complex, stressful, and sensitive task, driving can be disturbed by various factors, from the health status of the driver to the environment in the vehicle. Recent studies have focused on driver’s behavior-related physical status, i.e., alertness, drowsiness and fatigue [10,11,12,13,14,15,16,17]. This is an important issue that must consider how to perceive the health status and the hazardous environmental situation and how to provide helpful services to the driver so as to prevent the incoming accident. However, recent studies have not provided a comprehensive and direct method for taking care of the driver’s health, as well as providing the appropriate services for safe driving. Therefore, the healthcare service system that can monitor multiple vital signs through various sensors during driving and provide appropriate notification, recommendations, and even possible off-line emergency services is highly desirable for elderly drivers or drivers suffering from chronic diseases. By taking advantage of the sensing infrastructure and technologies provided by smart cities, more intelligent health services can be achieved to coordinate existing resources (information or services) to satisfy the personalized requirements of the drivers according to the analysis of real-time sensory data, which can be considered as smart health (s-Health) [18].

To address the need for tracking the health status of drivers and providing smart personalized services, we designed a context-aware smart health service system that continually collects real-time sensory data and proactively provides appropriate services for the driver to make them aware of the hazardous situations. Vital signs including blood pressure, heart rate, body temperature, and blood sugar are monitored to assess the health status of the driver. Environmental parameters are collected to observe the in-vehicle environment conditions, including carbon monoxide (CO), oxygen, and temperature, in case of CO poisoning, hypoxia and heatstroke (or hypothermia), respectively. To depict the driver’s situation, an ontology model for the sensor network in the vehicle is proposed in which the sensors, the driver, the vehicle, and the observation process are defined and correlated. Several types of reasoning rules are defined with different purposes for the service system to draw the health status from the sensory data. The assessment model for evaluating the observed result is proposed to provide an overall health status at the time. The provision of the business services is demonstrated with regards to different hazardous situations.

The rest of the paper is organized as follows: Section 2 gives an overview of related works in m-Health, driver status detection, and s-Health. Section 3 briefly describes the architecture of the proposed system from the collection of the sensory data to the provision of the business services. In Section 4, the ontology-based context model for the semantic sensor network in the vehicle is described and the design of the reasoning rules is discussed to decentralize the observed sensory data. The assessment model of the observed status and the provision of customized services are introduced in Section 5. Section 6 reports the evaluation results and Section 7 concludes the paper.

## 2. Related Work

In the m-Health scenario, vital signs, including electrocardiogram (ECG/EEG), heart rate, respiratory rate, blood pressure, blood sugar, and temperature, can be measured and obtained from wearable sensors in a nonintrusive way. The patient’s data, including symptoms and vital signs, is collected by the mobile device through User Interface (UI) interaction or directly from sensor devices. The medical recommendation or alerting services can be provided when the conditions of the patients deteriorates. In [7], a healthcare service system is proposed in which the patient’s vital signs are acquired from the sensors and then visualized as line charts for the patients, relatives and physicians. These vital signals are simply collected and exhibited without further analysis. Tele-monitoring of the patients’ real-time activity or chronic conditions has been implemented in recent studies. The vital signs and general symptoms are obtained from the report of the patients, and then the collected data are calculated into risk scores through basic analysis. The final result is delivered to medical staff for assistance and to the patient for notification [8]. A home-based continuous care system is proposed to provide alerting or notifying services for relatives or medical staff with regard to the reasoning on the sensory data acquired from biomedical and environmental sensors [9]. The sensory data is analyzed according to the context reasoning which determines the occasion to trigger the alarm.

Driving is a complex scenario that is sensitive to the activities of the driver. Recent studies pay attention to the driver’s behavior-related physical status, i.e., alertness, drowsiness, and fatigue. The ECG/EEG and the extracted heart rate (HR) are measured through the nonintrusive equipment and taken as the indicators of the driver’s cardiac health state [10,11,12,13]. Visual sensors (cameras) are widely utilized as a nonintrusive method by extracting the facial feature or the eye movement to monitor and recognize vigilance or fatigue during driving [14,15,16,17]. A smartphone-based driver drowsiness monitoring system has been proposed to detect the driver’s fatigue during driving [15]. The heart rate variability, blood pressure, temperature, speed, and percentage of eyelid closure are integrated to predict and analyze the driver’s vigilance index. The data are collected from sensors deployed on the vehicles through a Bluetooth connection and transferred to the mobile phone. To alert the driver’s attention, a fake call service is implemented and will be triggered when the evaluation metric reaches the threshold.

As the context-aware complement to m-health within smart cities, s-Health allows the extension of the coverage of health from hospitals and adapted homes to everywhere in the city [18]. By leveraging the sensing capabilities of smart cities, some smart health applications have been proposed in recent studies. A smart route planning application is proposed for the patients with respiration problems to find the route to their destination with a minimal effect on their health. In the proposed system, the sensors are deployed to detect air pollution and pollen in the city to obtain the measurement data for route planning [19]. Based on the concept of s-Health, an emergency response system is implemented to report and respond to emergencies of motorcycle riders. With various sensors deployed on the motorcycle, the system can detect the accidents of the riders and assign the nearest ambulance to them. Moreover, the traffic lights can be arranged to save time for the rescue [20]. A smart space-based approach is introduced for implementing the smart health applications. In the proposed approach, with the aim of delivering the in-hospital services to the remote patients, some certain intelligence attributes are emphasized including service ubiquity, context-awareness, and service construction [21]. The wireless communication between ambulance and urban deployed access points has been analyzed in a segment of an urban scenario. The received power levels and time domain parameters of the transceivers with different locations have been estimated to assist the deployment of the access points in smart cities [22].

As the critical factor of safe driving, the driver’s health status and environment factors should be carefully considered. However, to the best of our knowledge, the issue in regard to the smart health services for drivers seems not to have been extensively investigated by the related work.

## 3. System Architecture

**Figure 1:**
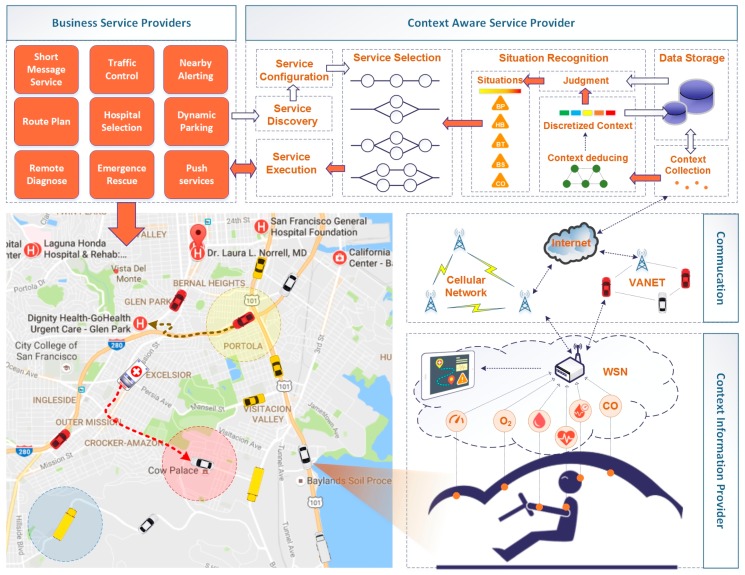
System architecture consists of four main parts Context Information Provider (CIP), Commutation, Context-Aware Service Provider (CASP) and Business Service Provider (BSP).

The system architecture shown in Figure 1 consists of several participants including Context Information Provider (CIPs), Communication Providers (CPs), Context-Aware Service Providers (CASPs) and Business Service Providers (BSPs). Each of these involved providers plays important roles in the m-health service system. The context information of the drivers is generated and uploaded to the service system through heterogeneous networks. By processing, deducing, and assessing the contextual information, the situation of the driver can be recognized, which can direct the selection and execution of the necessary business services for the driver.

### 3.1. Context Information Provider (CIPs)

As the foundation of the m-health service system, the CIPs provide plenty of data acquired from radio-frequency identification (RFID) readers, various sensors, embedded systems, legacy information infrastructure, etc. The CIPs are responsible for the production of the context information and keep improving the cost efficiency (especially on the deployment of the sensors) and QoS of the data acquisition (accuracy, reliability, timeliness, integrity, etc.). With large numbers of sensors deployed, CIPs continually monitor and generate the key raw context of the drivers, mainly including the vital signals and environment variables of the vehicle. With the development of the sensor techniques, sensors are becoming more powerful, cheaper and smaller in size. These sensors provide more effective and feasible methods to detect the blood pressure, heart rate, body temperature, etc. The wireless communication technique can connect, gather and manage the spatially distributed cluster of sensors in the forms of a Wireless Sensor Network (WSN) such as Body Area Network (BAN) or Vehicular Sensor Network (VSN). With an onboard terminal as the gateway node of the WSNs, the sensory data can be gathered, processed, and encapsulated into data-interchange formats such as XML or JSON for further interaction.

### 3.2. Communication Providers (CPs)

Ubiquitous connection is sustained by the CPs which provides the transmission of the context and the services pushed to the drivers. Traditional wireless communication methods including WLAN standards (802.11a/b/g/n) and cellular networks (e.g., GPRS/3G/4G/5G) bring a convenient and direct way for data interaction. As an emerging communication solution, the 802.11p protocol-based Vehicular Ad hoc Networks (VANETs) in Vehicle-to-Vehicle (V2V) and Vehicle-to-Infrastructure (V2I) schemes can also be provided as supplemental network resources for data transmission, especially when lacking communication infrastructure (out of coverage area) or in poor conditions (heavy overload).

### 3.3. Context-Aware Service Provider (CASP)

The raw context information of the driver is uploaded to the CASP, which is responsible for the fusion of the context to provide the driver with an appropriate business invocation policy according the occurring situation. The CASP consists of modules working on context deduction, situation assessment, service policy configuration, and business service execution. Raw context is first processed in the context deduction module based on the context model and reasoning rules to produce the observed status of the driver. On the basis of the historical observed status in a finite time, the situation of the driver can be detected in the situation assessment module. The service policy is the mapping between the driver’s situation and the available business services, which is configurable for the customized demand in accordance with the business scenario and the ability of the business services provided by the BSPs. This configurable business policy provides the loose-coupled feature as well as the reusability of the context information and discovered business services. The service execution module will invoke the business services in light of the service policy, which contains the execution schema of the service composition.

### 3.4. Business Service Providers (BSPs)

BSPs provide a variety of business services consisting of informational ones and functional ones. The information services answer the inquiry from the invoker and returns information (e.g., the hospitals around the driver, the specific hospital and the fastest route path to get there) as soon as possible. The functional services mainly focus on the procedure of the task, for example, pushing a message for notification. Another example for the functional services is the emergency rescue service, which provides online response (e.g., return the assigned hospital and dispatched ambulance) and offline service (direct the ambulance to the driver in danger in accordance with the driver’s static or dynamic location). With the development of cloud computing, the business services can be encapsulated into web services deployed in a service-oriented cloud computing architecture which brings the advantages of agility, lower cost, device independency, location independency, scalability, etc.

## 4. Representation and Reasoning of the Driving Context

In the driving context, the driver’s situation should be abstracted to include the driver’s health status and the inner environment of the vehicle. Blood pressure, heart rate, body temperature, and other vital signs constitute the health condition of the drivers. In addition to the vital signs acquired from the driver, the change of the environment in the vehicle (e.g., the temperature, CO, and oxygen concentration) can also have a critical impact on the driver’s condition. Against the backdrop of m-health application scenarios, an appropriate model is needed to indicate the concepts, sensory data, and the implied knowledge of the driving scenario. In the following sections, we describe the ontology model and the inference of the observed status over sensory data.

### 4.1. Vehicle-Driver Semantic Sensor Network (VDSSN) Ontology

To depict the driver’s context, the Vehicle-Driver Semantic Sensor Network (VDSSN) ontology is proposed in the Web Ontology Language (OWL) [23] and built in Protégé-OWL editor [24]. The VDSSN ontology is an embodiment and expansion of the Semantic Sensor Network (SSN) ontology developed by the W3C Semantic Sensor Network Incubator Group [25]. As a sensor domain ontology partly aligned with DOLCE Ultra Lite (DUL) [26], the SSN Ontology defines the capabilities of sensors and sensor networks with a description of the sensors, sensor observations, and related concepts to assist the management and querying of those sensor networks. The SSN ontology provides an extensible and flexible framework to describe the context information. Therefore, as a standard developed by W3C, the SSN ontology is introduced and expanded in this paper for the representation of the context information. The primary purpose of the VDSSN ontology is to classify things in terms of semantics and prepare for the description of the reasoning rules in the inference process. Moreover, the ontology model could enable knowledge reuse for various implementations and deployments without starting from scratch.

**Figure 2:**
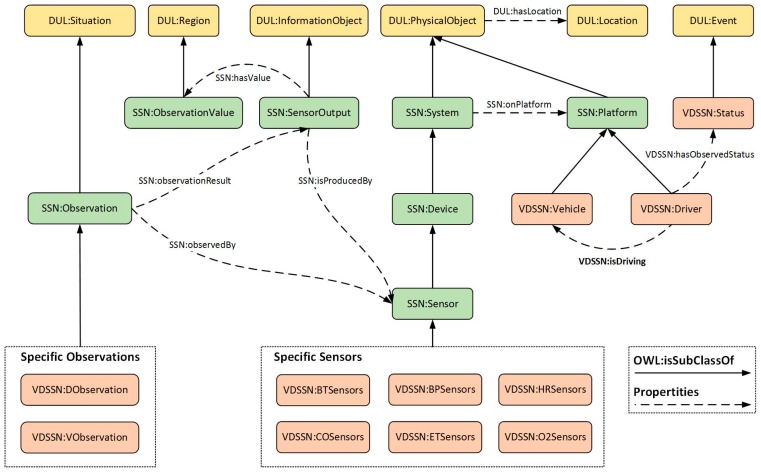
Inheritance relationship among Vehicle-Driver Semantic Sensor Network (VDSSN), SSN and DOLCE Ultra Lite (DUL) ontologies.

Part of the VDSSN is shown in Figure 2. According to the inheritance of the SSN and DUL upper ontology, the features of the semantic sensor network can be achieved. To describe the m-health context with minimal extension, some essential entities are appended to augment the representation of the driver and the vehicle. In the VDSSN ontology, some entities are inherited from the upper ontologies, including *Sensor*, *SensorOutput*, *Platform*, *Observation*, *ObservationValue*, etc., as well as corresponding properties (e.g., *hasValue*, *observationResult*, *isProduceBy*). The Platform entity is further extended into subclasses, i.e., *Vehicle* and *Driver* with the *isDriving* property to describe the relation between them. As the indication of the changed situation, *Status* is defined as a subclass of *Event* from DUL and attached to the driver with the *hasStatus* property. Various sub-properties of *hasStatus* can be defined for the description of different situations, e.g., *hasBTStatus* to indicate the blood pressure status, and *hasETStatus* to represent the temperature status in the vehicle. *DObservation* and *VObservation* describe the observation process on the driver and the vehicle and are further divided into specific instances according to the function of the sensors. The VDSSN ontology can be expanded under practical demand.

**Figure 3:**
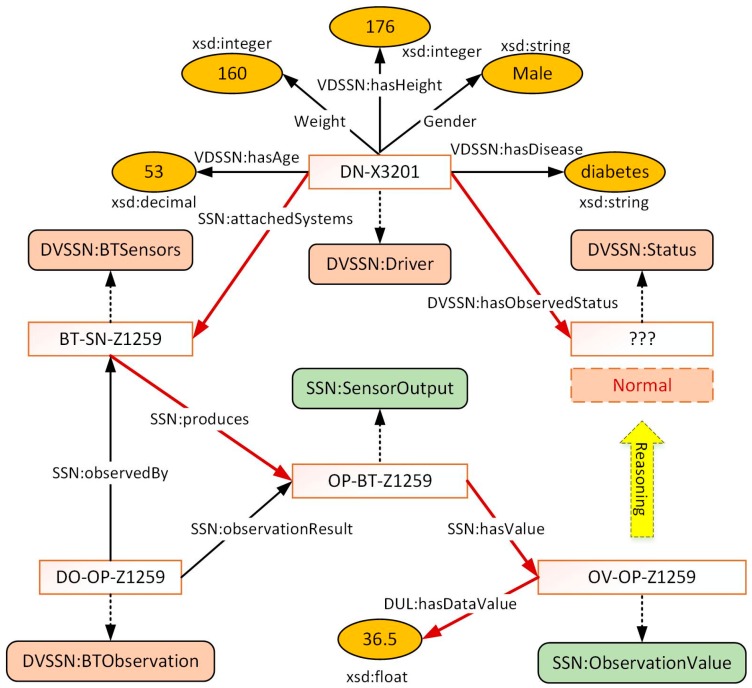
Demonstration of the relationship between instances.

The VDSSN ontology provides the skeleton frame to define the concepts and data among the domain of m-health which can be further utilized for the extraction of implied knowledge (i.e., context reasoning). In the ontology, data are presented as instances of concepts, and can be generated and connected through properties (i.e., object property and data property). For example, the driver’s profile, such as age, weight, and medical record can be represented with regard to *hasAge*, *hasWeight*, and *hasDisease*, respectively. Exhibiting directly related concepts and properties, Figure 3 shows the scenario in which a driver being observed by a body temperature sensor attached to him. The driver with identifier “DN-X3201” is attached by sensor “BT-SN-Z1259” which observes his body temperature with observation process “DO-OP-Z1259”. The sensor “BT-SN-Z1259” has a corresponding output “OP-BT-Z1259” with an observation value “OV-BT-Z1259”. The acquired data value “36.5” is a float data type with regard to the XML Schemas Definition (XSD). The instances of the driver’s status are enumerated from 0 to 3, which refer to the degree of the observation result. As the high-level implied context, the driver’s status cannot be obtained directly from the sensors. In light of the relationship and pre-defined rules, the driver-related context can be retrieved along with the clues built by the properties, and the status can be determined from context reasoning. The process is identified with the red lines. Further detail of the reasoning process is described in Section 5.

### 4.2. Acquisition of Observed Status

Residing at the heart of the service system, the context reasoning subsystem is in charge of deducing high-level context. The collected context is first interpreted in accordance with the VDSSN ontology in order to be discernible for reasoning. Then the reasoning module is triggered to get access to all related resources to coordinate appropriate instances to obtain the observed status of the driver in the present observation.

With regard to the observed sensory data and reasoning rules, high-level context is generated as the indicator of the observed result. There are two main purposes of the context reasoning module. One is to decentralize the observation value for context division. For example, the driver’s age can be divided into four sections, which are “<44”, “44–54”, “54–64” and “≥64”. The other is to assess the observed status according to the related raw instances and decentralized context. Blood pressure, body temperature, heart rate, blood sugar and other vital signs are important indications of the driver’s health status. Under normal circumstances, these vital signs remain in a stable range and vary according to the behavior (e.g., strenuous movement, long fasting) or mood (e.g., anger, anxiety, despair) of the person. However, the extravagant excess of the bearable range can put the driver an extreme situation (for instance, great pain, lose conscious, uncontrollable body) which is very dangerous for safe driving. In addition to the vital signs, environmental variables, such as CO, oxygen, and temperature in the vehicle, are also critical for the driver’s status, which may cause CO poisoning, anoxia, and heat stroke, respectively.

This paper adopts ontology-based reasoning and user-defined reasoning for the deduction of high-level context with the eventual aim to achieve a comprehensive definition of the driver’s status. Ontology-based reasoning (i.e., OWL reasoning) is responsible for the specification of the terminological hierarchy. With regard to the restricted relations, cardinality, attributive character, etc., the consistency of instances (e.g., disjoint), inclusion relationship (e.g., sub class of) can be clarified. For example, assuming an onboard system “SYS-OB-EU208” has an *onPlatform* property related to driver “DN-3201”. Then, as a subclass of “SYS-OB-EU208”, the property between sensor “BT-SN-Z1259” and driver “DN-3201” can be inherited as *onPlatform* through ontology-based reasoning. User-defined reasoning is discussed below in detail.

**Figure 4:**
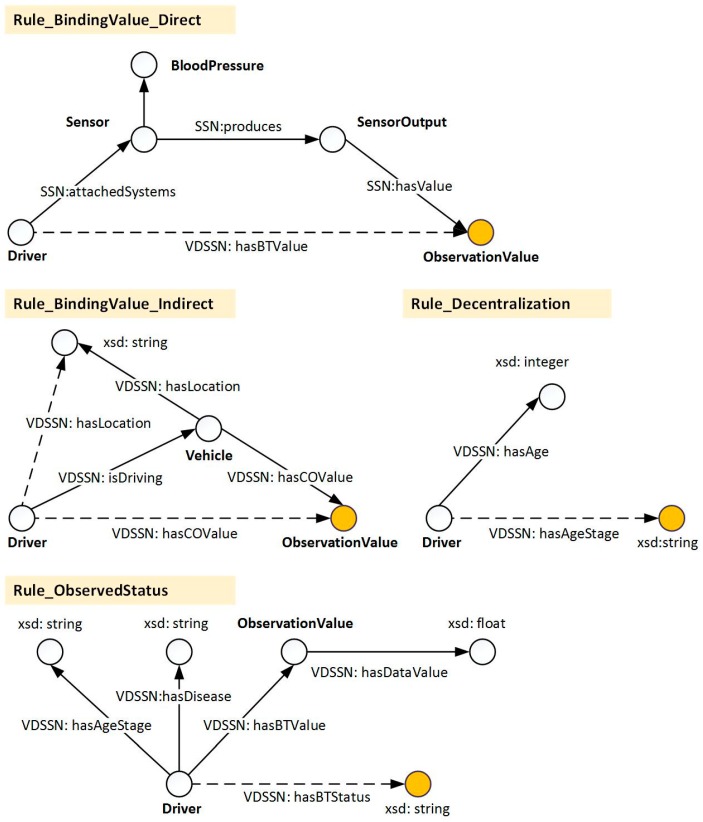
Demonstration of the reasoning processes.

As the backbone of the reasoning process, user-defined reasoning provides a more flexible way to determine the high-level context. With regard to different purposes, the rules are divided into four categories, i.e., value directly binding, value indirectly binding, value decentralization, and observed status determination. The graphical representation of these reasoning processes is shown in Figure 4. The division of the rules can decouple the rules and enhance the flexibility of the rule configuration. Furthermore, the efficiency of different rules can be evaluated and improved separately. The directly binding value is in charge of binding the observed data value (e.g., blood pressure value) to the observed entity (i.e., driver and vehicle). The indirectly binding value refers to the data bound from another platform in light of the property between the platforms, e.g., *isDriving*. To easily integrate this with other rules and reduce the complexity of rules, continuous data (e.g., age) needs to be partitioned according to the decentralization rules. Finally, the driver’s observed status determination is in charge of the final decision of the driver’s observed status based on all coordinated resources in the present observation. This part of the rules consists of the health condition and situations that affect the environment. As a special kind of decentralization rules, the key of the observed status rules is to be based on the driver’s possible reaction (symptoms) to different vital or environment signs, then guide the decentralization of the corresponding observed values. The determination of the observed blood pressure status is taken as an example and discussed below.

Hypertension, the most common disease in primary care, may lead to myocardial infarction, stroke, renal failure, and death if not detected early and treated appropriately [27]. As shown in Table 1, according to the classification of blood pressure in the Seventh Report of the Joint National Committee on Prevention, Detection, Evaluation, and Treatment of High Blood Pressure, hypertension was defined as a BP of 140/90 mm·Hg or higher, which starts as the stage 1 hypertension while the normal range of systolic BP (SBP) is less than 120 mm Hg and diastolic BP (DBP) is less than 80 mm·Hg [28]. Although not a category of JNC, an SBP > 180 mm·Hg or a DBP > 120 mm·Hg is considered as a “hypertensive crisis” and can further lead to “hypertensive urgency” or “hypertensive emergency” with severe symptoms (e.g., headache, shortness of breath, unconsciousness) [29], which can have a serious impact on safe driving and pose a significant threat to lives. As a matter of fact, blood pressure may increase along with the age according to the research on the mean blood pressure in different age groups [30], which means the BP of older people is often higher than that of younger people.

With regard to the definition, classification, and symptoms of hypertension, the preliminary factors for the status rules can be identified as age, disease (specific to the particular severity of hypertension), and observed BP values. The division example of the observed SBP status is shown in Table 2 with four stages. The reasoning module is implemented with Jena2 [31] and some of the typical rule instances are shown in Table 3. In addition to the blood pressure, other rules for the observed hazardous state include body temperature, heartbeat, CO concentration, and in-car temperature can be defined and affiliated into the reasoning module.

The driver’s health status is difficult to represent due to the extremely complex relationship between vital signs, symptoms and diseases. With physicians and medical data involved, specific reasoning rules can be designed to correlate various vital signs, physical traits and medical records to represent various hazardous situations with different symptoms and diseases. Further adjustment to the rules by a physician is needed to match specific requirements of real cases and for precise identification of situations.

## 5. Context-Aware Health Care Service

### 5.1. Situation Assessment

In a real-world scenario, due to the sensor sensitivity, human factors and instantaneous data jitter, the current observed status cannot be arbitrarily trusted or taken as the only decisive factor to represent the driver’s status.

Currently, the method to deal with acceptable abnormal situations has not been considered in existing context-aware solutions, such as the application in a smart home [32] or for remote healthcare [9]. Providing services without checking the observed result can cause distraction from safe driving and increase the burden on the service system.

As a critical supplement, historical observed results can be provided as assistant diagnostic information for the assessment of the driver’s condition. To filter the observed status, this paper proposed a high level assessment model to determine the situation based on the historical observed status. For each observed situation *i* (e.g., SBP, body temperature), let $S_{i}$ denote the set of assessment statuses of the observed situation *i*:

(1) $$S_{i}\;=\;\left\{s_{t_{1}}^{i}{,\;s}_{t_{2}}^{i}{\;,\text{…},\;s}_{t_{n}}^{i}\right\}$$

where $S_{t}^{i}$ is the assessment status at time *t*, and $t_{n}$ denotes the current time. The set of observed statuses at each acquisition time is denoted as $V_{i}$:

(2) $$V_{i}\;=\;\left\{v_{t_{1}}^{i}{,\;v}_{t_{2}}^{i}{\;,\text{…},\;v}_{t_{n}}^{i}\right\}$$

in which $V_{t}^{i}$ is the observed status at time *t*. Let *T* denote the time range in which the reasoning results are active. Then the set of valid observed statuses which has same status as the current one is represented as follows:

(3) $$G_{i}\;=\;\left\{v_{t}^{i}{|v}_{t}^{i}=v_{t_{n}}^{i},\;(v_{t_{n}}^{i}\cdot time\;-\;v_{t}^{i}\cdot time)<T,\;v_{t}^{i}\;\in {\;V}_{i}\right\}\;\cup \;\left\{v_{t_{n}}^{i}\right\}$$

Let *H* denote the sample frequency, and to a given assessment parameter λ Є (1T·H, 1], the valid quantity of the observed status can be indicated as E = T·H·λ. Then, the assessment status of the current situation $s_{t_{n}}^{i}$ is determined as:

(4) $$s_{t_{n}}^{i}\;=\;\{\begin{array}{c} s_{t_{n-1}}^{i},\;\left|G_{i}\right|<E\\ \;v_{t_{n}\;}^{i}\;,\;\left|G_{i}\right|\geq E \end{array}$$

Especially, the assessment status at the very beginning will be set as normal, which can be denoted as $s_{t_{1}}^{i}\;=\;0$.

**Figure 5:**
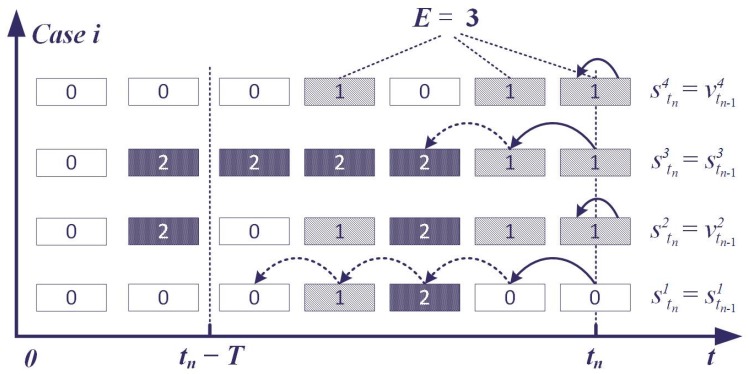
Cases of situation assessment process based on historical status.

The observed status can be filtered with regard to the advantage of the buffered observed status, which takes the historical data as an important factor to assess the current situation. Some of the assessment cases are shown in Figure 5. In *Case 1*, the assessment status is determined as 0 and not disturbed by the sudden increase of the observed status. *Case 2* demonstrates the change of the assessment status when $\left|G_{i}\right|\;>E$. In *Case 3*, at the beginning of the change, the observed status is buffered and the current status stays the same as the former one (status code is 2).

The continual and alternate changing of the observed status needs to be focused upon because it can trigger the service invocation too frequently and increase the burden on the service system. Meanwhile, the driver may be disturbed with repeated information and this could pose a threat to safe driving. With regard to the alternate observed status, the higher status between the former assessment status $s_{t_{n-1}}^{i}$ and the current observed status $v_{t_{n}}^{i}$ will be taken as the current status when the $\left|G_{i}\right|$ reaches E. The following equation describes the interception of eligible observed statuses to avoid the unnecessary alternation of the assessment status.

(5) $$s_{t_{n}}^{i}\;=\;\{\begin{array}{c} s_{t_{n-1}}^{i}\;,\;\left|G_{i}\right|\;=\;E,\;v_{t_{n}}^{i}\;\leq {\;s}_{t_{n-1}}^{i}\\ v_{t_{n}\;}^{i}\;,\;\left|G_{i}\right|\;=\;E,\;v_{t_{n}}^{i}{\;>s}_{t_{n-1}}^{i} \end{array}$$

**Figure 6:**
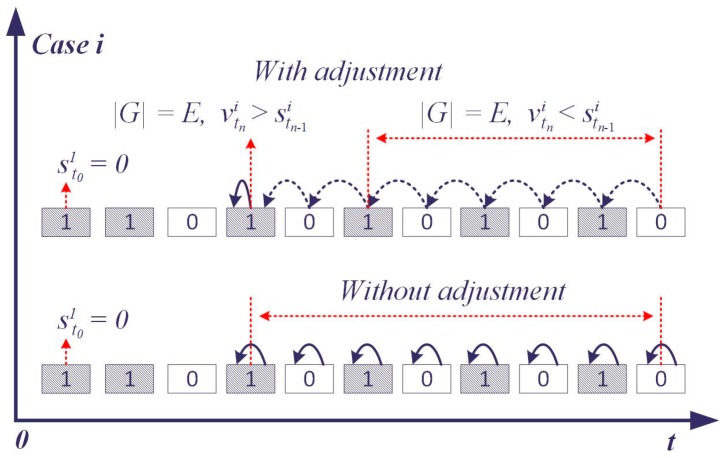
Adjustment of the assessment status.

Figure 6 shows the adjustment of the assessment status when $\left|G_{i}\right|$ reaches *E*. The former assessment status indicates the overall situation in T, and a higher former assessment status is assumed that it has a higher influence on the driver when the valid quantity of the observed status is right on the edge ($\left|G_{i}\right|\;=\;E$). In this context, the current observed status is buffered and the current assessment status will not change until $\left|G_{i}\right|$ exceeds *E*.

### 5.2. Providing Customized Services

The key of the context-aware service system is to provide the driver with appropriate services with regard to the assessed health status. The assessment health status indicates the severity of the abnormal situation, when the driver may have a different capacity to react. According to the severity of the situation, it can be divided into a controllable situation and an uncontrollable situation. In the controllable situation, the driver is conscious of the notification and able to eliminate the hazardous situation by following the instructions themselves. An uncontrollable situation means the driver is incapable of taking action (lost control of the body) to protect themselves from the critical condition (e.g., heart attack, stroke, hypertensive crisis). For example, when the driver’s systolic blood pressure is assessed as dangerous, the driver may feel uncomfortable and at certain risk of suffering symptoms such as a headache or shortness of breath, which could lead to an accident. However, in this situation it can be possible for the driver to take proper actions to return to the normal condition for safe driving. If the situation is out of control, appropriate services can be recommended for the driver to choose.

**Figure 7:**
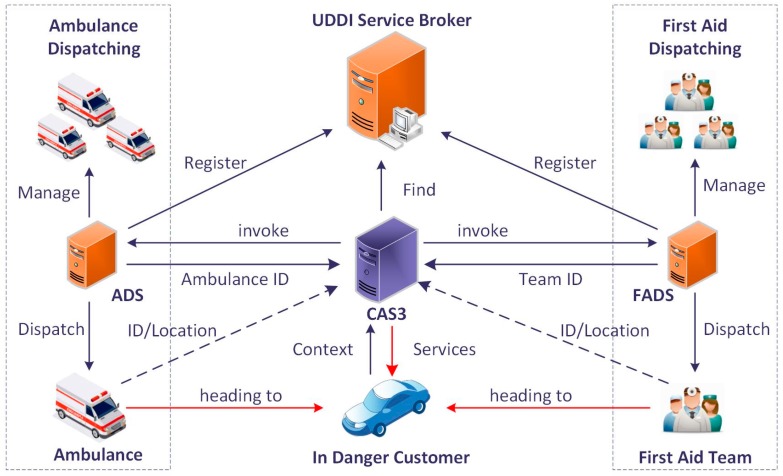
The invocation of selected services.

In the Service-Oriented Architecture (SOA), services are implemented and encapsulated into web services. With regard to the registration to a UDDI service broker, the services can be published and seen by consumers. These business services provide functions or information for the service system or the drivers. The functional services are Information Pushing (e.g., short message service, push notification service for mobile equipment), and online services with offline actions which offers the driver emergency rescue services. For example, the ambulance dispatching service may receive the driver’s current status, location, and other critical information to assign the nearest available ambulance to the driver. The information services execute the query task to provide information on location selection, path planning (with traffic conditions), etc. The Onboard Alarming Service (OAS), Route Selection Service (RSS), Ambulance Dispatch Service (ADS), and First Aid Dispatch Service (FADS) are taken as an example to demonstrate the provision of the services in the Context-Aware S-Health Service System (CAS3). As the carrier of some services, the push service is not involved in order to simplify the situations. The OAS is responsible for alerting the driver to the abnormal situation through an audio alarm. RSS can provide the appropriate route path to a given location with regard to the traffic condition. The ADS dispatches the nearest ambulance for the customer from the available ambulances in a certain nearby area. The FADS provides the driver with first aid services provided by a nearby clinic, community sanitary service center or volunteers. FADS can be a supplementary service if the ADS system is not available or the dispatched ambulance is too far away. The invocation process of the ADS and FADS are shown in Figure 7.

Service policies are sets of available services permuted and combined into compositions with different purposes. According to the emergency level of the health status from Warning to Extreme Danger, the service policies can be designed and configured in a service configuration module to choreograph the appropriate business services. In order to adapt to different scenarios (controllable or urgent), for each policy there exists a configured interaction mode (*Rec*/*Enf*) to constrain the service execution with or without the user’s acknowledgement. As shown in Table 4, the service policies can be configured and customized in the service configuration module. The service quality defines the restriction of the provided services including the types, forms and combination of the services.

In light of the assessment status, the invocation of the selected composition will be triggered when the status is altered. The services in the selected composition will be executed according to the order and interaction mode. For the *Rec* mode, the service is recommended to the driver and not executed until the service is confirmed. If the service is executed, the results from services, including the route path, and assigned ambulance will be incorporated as a continuous service to the user until the process is canceled or finished (e.g., the driver arrives at the recommended hospital, the ambulance contacts or meets the driver).

The following example takes the abnormal systolic blood pressure status as the demonstration scenario, in which it is assumed that Tom is driving a truck and his health and the in-vehicle environment indicators are acquired and collected every 10 second. Supposing that his last assessment status of systolic blood pressure is WARNING and his current SBP status is DANGER (165 mm Hg) after the assessment, and then, with regard to this alteration, the service system queries the service policies for this changed context and selects the service composition (OAS→*Rec*(RSS)→*Rec*(ADS→FADS)) and executes it for the driver. Then the route plan service and emergency rescue service will be invoked in *Rec* mode. The driver will receive the notification on the terminal to choose the services he needs or just to ignore the notification and continue. If the emergency rescue service is confirmed, the nearby ambulance and the first aid team will be requested, assigned, and guided to the driver. Meanwhile, the rescue services return the client identifiers of the assigned ambulance and the first aid team. The service system will assign the ambulance and the first aid team to the driver, and then keep pushing the location of the driver to them until they meet the driver in danger. Moreover, the services will be enforced when the situation becomes extreme. On that occasion, the service policy (OAS→SMS→RSS→ADS→FADS) will be selected and executed for the driver without confirmation.

## 6. Evaluation

The service system is evaluated in an experimental environment with an Android-based ARM 11 platform (Real6410) as an onboard terminal and a PC (Intel Core 2 Duo processor, 2.94 GHz, 4G memory, Lenovo, Beijing, China) as a context-aware service system server. The terminal is embedded with GPS and GPRS (MC52I, 4G cellular network) to acquire the location and transport the sensory data to the server, respectively. The client is developed as an android application which is responsible for the collection of the sensory data.

In the experiment, the sensory data is simulated and generated on the client with a designed trend and local random value at each stage. The sensory data is sent to the context server and transformed to the context data by a context interpreter, which is responsible for creating the instance of the entities upon the VDSSN ontology. The ontology model is kept at a minimum scale to support the representation of the driving situation. Reasoning rules are designed, including: seven direct binding rules, three indirect binding rules, four decentralization rules (for age division), and 96 rules for status determination.

The experiment condition is conducted as follows: (1) We take a single onboard terminal to generate and transport the sensory data including body temperature, blood pressure, blood sugar, heart rate, CO concentration, O_2_ concentration and in-car temperature; (2) The sample frequency *H* is set to two times per minute; (3) The valid time windows *T* is conducted as 2.5 min.

**Figure 8:**
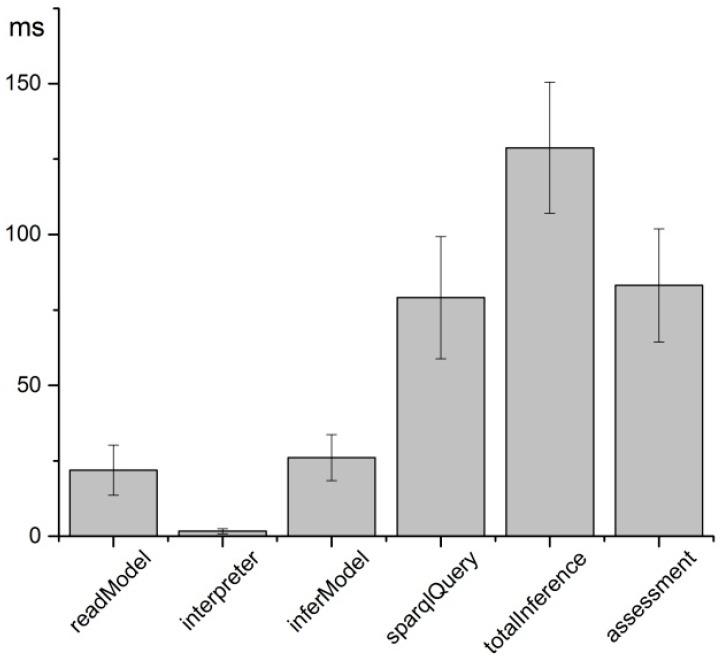
Time consumption from the sensory data to assessment status.

To evaluate the time consumption of the service system to perceive the situation based on the sensory data, seven types of sensory data are processed one hundred times. Figure 8 reports the mean and standard deviation values in milliseconds during the reasoning and assessment process for every sampling period of the sensory data. The *readModel* represents the time consumption for reading the ontology model for further reasoning. It takes 1.66 ms on average for the context interpreter to transform the sensory data into context data, including creating individuals of the classes and adding properties between them. Then the set of RDF data is bound to the inference engine to create the inference model (*inferModel*). The observed status is acquired as the reasoning result by means of SPARQL querying the *inferModel*, which takes 79.1 ms on average for seven queries in each round, in order to acquire the observed status of blood pressure, body temperature, CO concentration, etc. The mean time consumption of the total reasoning process (*totalInference*) is 128.7 ms. It takes 83.14 ms to assess the status of the driver in seven types of situations. The total time consumption from receiving the sensory data to acquiring the assessment status is, on average, 340.6 ± 47.8 ms.

**Figure 9:**
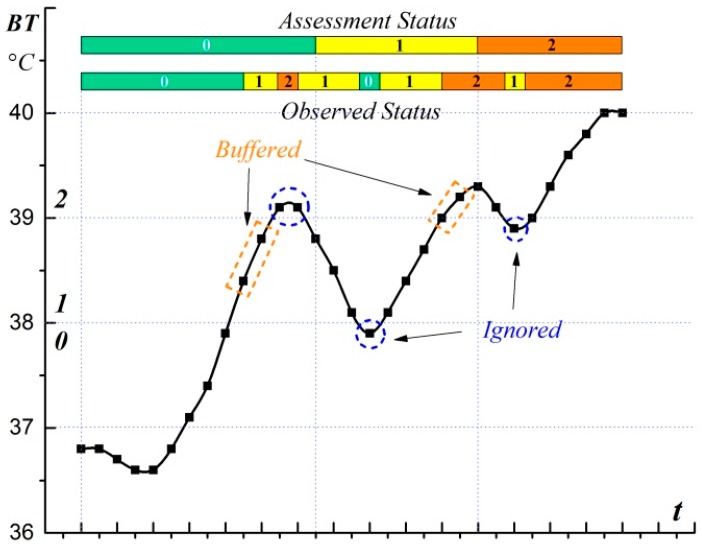
Assessment demonstration during the observation of body temperature.

**Figure 10:**
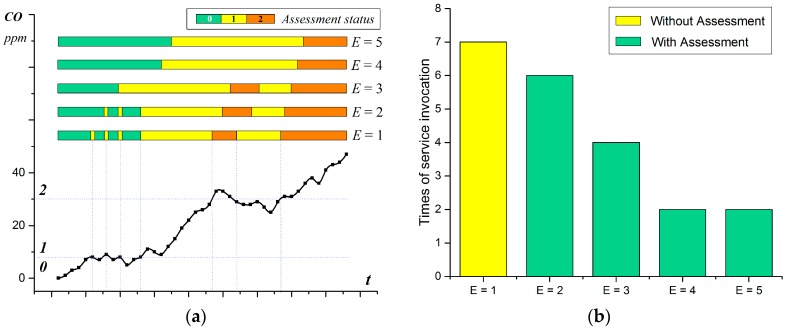
Assessment result as the valid quantity increases from 1 to 5 during the CO observation: (**a**) Assessment status; and (**b**) Times of services invocation.

The assessment of the body temperature status is taken as a demonstration of the assessment process, in which the threshold value of statuses 1, 2 and 3 are 38.0, 39.0, and 42.0, respectively. The divisions of the body temperature are designed as four rules for the inference of observed body temperature status. The parameter λ is set at 0.6; thus, the valid quantity *E* = 3, which means for every changed observed status, only if at least two valid historical observed results acquired in 2.5 min are the same as the current one, can the assessment status change along with the observed one. Figure 9 shows the assessment process during the observation of body temperature in which the observed value keeps climbing with fluctuation. On the climbing side, when the threshold is first exceeded, or validates number of observed status $\left|G_{i}\right|\;<E$, the assessment status will not change (remain silence, as shown in orange rectangle) until $\left|G_{i}\right|$ is up to *E*. With regard to the buffer of the observed status, the sudden breaking of the threshold (rise or sink) with a quick return are ignored (the peaks in blue circles). Thus, the situation assessment transforms the intermittent situation into a relatively continuous status.To evaluate the effectiveness of the assessment model, the assessment of the CO status is taken as an example, in which the threshold value of status 1 and 2 are set to 8 ppm and 30 ppm, respectively. The experiment is repeated five times in which the assessment parameter λ is set as 0.2 (*E* = 1), 0.4 (*E* = 2), 0.6 (*E* = 3), 0.8 (*E* = 4), and 1 (*E* = 5), respectively. The assessment result of the CO status is shown in Figure 10a, which varies according to the valid quantity *E*. The assessment result is the same as the reasoning result when *E* equals to 1. With the increase of *E*, the beginning of the alteration keeps postponing and some of the peaks are tolerated due to the valid total observed status cannot reach *E*.

Figure 10b compares the execution times of the selected services in two conditions, by using the Situation Assessment and not using the Situation Assessment (the solutions proposed in [9,32]). The result indicates that the tolerance of minor fluctuations can reduce the number of service invocations. The services need to be invoked seven times without the Situation Assessment due to the existence of seven alterations of the observed status (as shown in *E* = 1), and the service system needs to respond to every single change of the observed status. In the condition with the Situation Assessment, the number of service invocations is reduced as the threshold λ increases (decrease to 2 when *λ* up to 0.8). The Situation Assessment helps the system to be tolerant to the acceptable abnormal situations (the observed status with $\left|G_{i}\right|$ less than *E*), and improves the service execution process.

The main purpose of the assessment model is to reduce the impact of the temporary alteration of the observed status on service invocation. By calculating the ratio of occurrence times of the current reasoning result to the total sampling times in *T*, the current status of the situation can be determined according to the parameter *λ*. The experimental results indicate the effectiveness of the assessment model with which the temporary alteration of the observed status in *T* can be tolerated without triggering the service invocation, which can ease the burden of the service system and avoid the distraction while driving. Moreover, the historical observed status acquired from the reasoning parts can also be utilized and take part in the assessment of the situation in *T*.

The time window *T* and parameter *λ* should be designed with regard to the practical application scenarios. As a crucial parameter in the assessment model, *λ* controls the strictness of the assessment process, i.e., decides when to accept the alteration of the observed result and trigger the services invocation. The decision of *λ* depends on the use cases. The bigger the *λ* is, the stricter the assessment process will be but the more time it will take for the system to respond. The invocation of the service responding to the normal alteration (with a stable observed status) can be postponed for (T·H·λ−1)·1/H. The time of such postponement is positively correlated to the valid time window *T* and parameter *λ* at a certain sample frequency *H*. In most use cases, this delay can be accepted when *T* is small (for instance, less than 5 min). For example, the time delay is one minute when *T*, *H* and *λ* are set as 2.5 min, two times per minute and 0.6, respectively. The default value of *λ* can be chosen in the range [0.6, 0.8], in which the effect of the assessment process and the time delay of the service invocation are acceptable (as shown in Figure 10b). The driver’s response to the provided services can also be utilized to adjust the assessment process dynamically and adaptively. For example, the value of *λ* can be gradually increased when the driver rejects the recommended services too frequently. Meanwhile, the service response rate to the altered observed status (the ratio of service invocation times to the occurrence times of the altered observed status) can be considered to restrict *λ* in a certain range (for example, keep the response rate above 60%). Moreover, to reduce the impact of the time delay on service invocation, some services with less overhead (such as notification services) can be invoked to notify the alteration of the observed status, and the rest services with large overhead will be triggered later when the alternation of assessment status is satisfied.

This paper has been primarily focused on the design and implementation of a context-aware smart health service system with the aim to detect the abnormal situations of the driver’s health status and provide customized services to meet actual demands. It must be noted that the proposed service system needs to continuously measure the driver’s vital signs. However, driving is a sensitive task that can be easily disturbed, which demands that the measurement process should minimize the impact on the driver as much as possible. To address this problem, some nonintrusive or wearable devices can be adopted to achieve the measurement of vital signs [33,34]. By introducing and optimizing various appropriate measurement methods, we believe the proposed system is feasible to perceive abnormal situations of the driver’s health status and guarantee safe driving.

## 7. Conclusions

With the increasing prevalence of chronic disease in drivers, there is an emerging need to provide the driver with continuous monitoring and customized services to ensure safe driving. In order to consistently monitor the health status and provide helpful services for drivers in case of various urgent situations, we propose a context-aware s-Health service system, in which it gathers the vital signs and environment parameters from the driver and his vehicle to provide the driver with customized services in light of the mapping between the assessment status and business service compositions. To alleviate the temporary fluctuation and reduce the unnecessary service invocation, an assessment model is proposed which exploits the historical reasoning results and comes to a conclusion regarding the status of the driver in a valid time slot.

Due to the complexity of the driving situation, the driver’s health status is difficult to represent with the various vital signs and environment parameters involved. The relationship among various vital signs reacting to various illnesses and environmental hazards is still the problem for future research to consider. Further work will pay more attention to the multi-sensor fusion model to assess the status of the situation more comprehensively and meticulously (e.g., obtaining the specific illness or symptoms from the sensory data for more accurate services).

## Figures and Tables

**Table 1:** Classification of blood pressure in JNC 7.

BP Classification	Systolic BP, mm·Hg		Diastolic BP, mm·Hg
Normal	<120	and	<80
Prehypertension	120–139	or	80–89
Stage 1 hypertension	140–159	or	90–99
Stage 2 hypertension	≥160	or	≥100

**Table 2:** Division example of observed systolic blood pressure status.

Age Stage	Disease	Status			
		0	1	2	3
44~54	Normal	<129	130–149	150–169	>170
44~54	Stage 1 hypertension	<130	140–159	160–179	>180
44~54	Stage 2 hypertension	<159	160–179	180–199	>200
54~64	Normal	<134	135–154	155–174	>175

**Table 3:** Typical rule instances.

Type	Reasoning Rules
Directly binding	(?a rdf:type mh:Driver) (?a attachedSystems ?b) (?b rdf:type SBPSensor) (?b produces ?c) (?c hasValue ?d) → (?a hasSBPValue ?d)
Indirectly binding	(?a, rdf:type, Driver) (?a, isDriving, ?b) (?b, hasCOValue, ?c) → (?a, hasCOValue, ?c)
Decentralization	(?a, rdf:type, Driver) (?a, hasAge, ?b) lessThan(?b, 44) → (?a, hasAgeStage, 0)
Status determination	(?a, rdf:type, Driver) (?a, hasAgeStage, 1) (?a, hasDisease, hypertensionS1) (?a, hasSBPValue, ?b) (?b hasDataValue ?c) ge(?c, 140) lessThan(?c, 159) → (?a, hasObservedSBPStatus, 1)

**Table 4:** Typical policies instances.

Assessment status	Service Quality	Policy Instances
Normal	None	None
Warning	Notification	OAS
Danger	Notification and Recommendation	(OAS→*Rec*(RSS)→*Rec*(ADS→FADS))
Extreme Danger	Notification and Enforcement	(OAS→SMS→RSS→ADS→FADS)
